# Spatial multiomics to inform immunocytokine engineering: knowledge base, gaps, and QC solutions

**DOI:** 10.3389/fimmu.2026.1852790

**Published:** 2026-07-15

**Authors:** Priyank Patel, Luke Daniel Batty, Marina Bleck, Shyam Nathan

**Affiliations:** Immunology & Respiratory, Boehringer Ingelheim Ridgefield CT USA, Ridgefield, CT, United States

**Keywords:** cytokine, immuno, omics, spatial, transcriptomic

## Abstract

Systemic pro-inflammatory cytokine therapies (e.g., IL-2) represented early milestones in immunotherapy, but their use is hampered by low response rates and severe off-target toxicity. In contrast, immunocytokines deliver cytokines directly to tumors, reducing systemic toxicity and enhancing efficacy. Spatial-omics provides deep insights into the tumor microenvironment (TME), enabling identification of druggable targets and accelerating development of novel antibody platforms and cytokine payloads. However, variability in patient sample quality affects data integrity, and platform differences require distinct preprocessing workflows. Spatio-temporal data demand spatial clustering to define disease-relevant niches, yet a lack of consensus about what constitutes a niche complicates interpretation and reproducibility. To overcome these challenges, effort needs to be made to improve sample collection and processing, and to reconcile the diversity of platforms with their technical limitations in niche identification. By combining knowledge of key TME cell types and marker expression with cytokines identified from autoimmune datasets, innovative immunocytokines can be designed to improve targeting, effectiveness, and patient outcomes.

## Graphical Abstract

**Figure:**
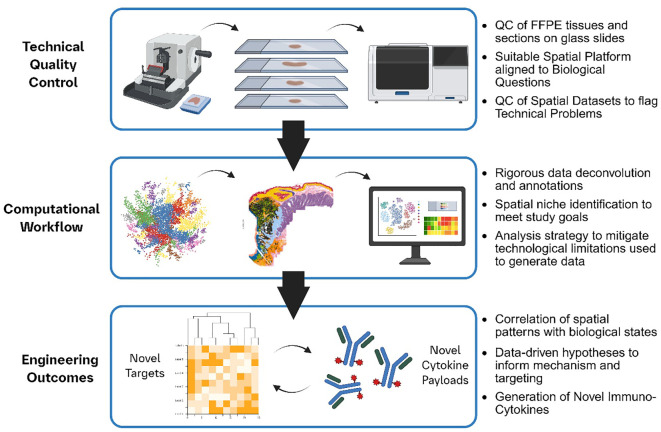

## 1 Introduction: spatial technologies and the role of cytokines in the TME

Tumors are highly structured and dynamic ecosystems composed of stromal, malignant and immune compartments that collectively drive tumor evolution by creating a localized cellular milieu or tumor microenvironment (TME). Tumor-associated cytokines operate in highly localized, heterogeneous patterns that critically shape immune cell recruitment, activation, and suppression within the TME. Bulk and single-cell RNAseq assays dissociate tissues, preventing *in situ* analysis of how immune states contribute to creation and maintenance of local TMEs. Profiling local TMEs using spatial multi-omics technologies, where cytokines such as TGFβ, IL6, CXCL12, and IFNγ exert distinct, region-specific effects on tumors and promote phenotypic plasticity, can enable identification of invasive or therapy-resistant states (1). Understanding where cytokines are produced and act is essential to interpret functional immune suppression, antigen presentation niches, and localized immunotherapy failure. As cytokine-targeted immunotherapies and combinations advance, spatial technologies are no longer optional tools but necessary platforms to identify actionable micro-niches, stratify patients by spatial immune architecture, and design interventions that overcome localized immunosuppression (2).

Spatially resolved molecular profiling technologies broadly fall into two major categories: spatial transcriptomics (ST) and spatial proteomics. The term *spatial transcriptomics* was introduced with the 2016 method that captured poly(A)+ RNA on spatially barcoded arrays and sequenced the resulting cDNA to reconstruct transcriptome-wide data (2). This foundational methodology was later commercialized by 10x Genomics as Visium, and subsequently refined in Visium v2, which improved sensitivity and incorporated more automated workflow steps. The Visium platform acquires transcriptomic data at *pseudobulk* resolution, constrained by its 55 µm circular capture spots arranged in a grid pattern. 10x Genomics subsequently developed Visium HD, 10x Genomics developed Visium HD, reducing the spot size to 2 µm and enabling near subcellular resolution (3). In parallel, imaging-based spatial transcriptomics technologies emerged, including Bruker CosMx (4) and 10x Genomics Xenium (5). Both platforms utilize barcode-based detection system to dectect mRNA transcripts and perform cell segmentation to achieve a sub-cellular resolution. MERFISH (developed by Vizgen) is another high-resolution imaging-based approach that employs combinatorial barcoding with sequential smFISH imaging to achieve high detection efficiency. Like CosMx and Xenium, MERFISH provides single-cell to subcellular spatial resolution but is constrained by targeted probe panels, typically capturing on the order of several thousand mRNA species (6). Detailed comparison of these platforms is provided in **Table 1**.

**Table 1:** Comparison of spatial transcriptomics platforms.

Platform	Company	Technology type	Detection	Spatial resolution	Dimensionality
Visium	10X Genomics	Sequencing-based spatial barcoding	Next generation sequencing	55 µm	Full Transcriptome
Visium HD				2 µm	
Xenium		Imaging-based multi-plex In-Situ hybridization	Padlock probes with rolling circle amplification	Single cell/Sub-cellular	Up to 5000 mRNA molecules
CosMx SMI	Bruker		Barcodes without any amplification		Up to >18,000 mRNA molecules
MERFISH	Vizgen		Barcodes with sequential fluorescence imaging		Up to 1000 genes

Recent benchmarking studies have systematically compared these commercial ST platforms, revealing important differences in sensitivity, specificity, resolution, and biological utility. 10x Xenium, Vizgen MERFISH, and Bruker CosMx were benchmarked on serial FFPE tissue sections across multiple tumor types. Xenium generated higher transcript counts per gene, and both Xenium and CosMx showed concordance with orthogonal single-cell RNA-seq data. All three platforms performed spatially resolved cell typing, though with varying false discovery rates and cell segmentation error frequencies (7). A recent study analyzed off-target mRNA probe binding and showed that probes designed for CosMx were more specific than Xenium. Cell segmentation performance represented another key differentiating factor among these platforms. Owing to its pseudobulk spot-based architecture, conventional Visium does not permit true single-cell segmentation. This limitation is partially addressed in Visium HD through replacement of discrete circular spots with a dense, continuous lawn of barcoded square features; however, accurate cell segmentation remains challenging with this tile-based acquisition strategy. In contrast, CosMx, Xenium, and MERFISH support true single-cell segmentation through the inclusion of membrane- or boundary-associated antibody staining. Nevertheless, substantial differences remain in the implementation, accuracy, and overall performance of segmentation workflows across these platforms (8). Collectively, these studies demonstrate that no single platform is universally superior, and the current approach seems to favor using a combination of a pseudo-bulk technology such as Visium or VisiumHD with a single cell resolution technology such as CosMx, MERFISH or Xenium.

Spatial proteomics platforms were similarly developed on multiple technological fronts. Both Multiplex-Ion Beam Imaging (MIBI) (9) and CODEX (10) use an imaging mass cytometry approach to detect between 50–100 protein markers simultaneously. During the MIBI protocol, antibodies are tagged with either a unique metal isotope followed by tissue rasterization and detection using a time-of-flight mass spectrometer. In contrast, antibodies are conjugated with a unique DNA barcode in CODEX that is subsequently imaged. These platforms enable simultaneous incubation of all antibodies avoiding the need for antibody stripping that may affect tissue quality with progressive cycles but are less flexible since the antibody conjugation process limits the diversity of testable clones for a particular target and is time consuming. Cyclic immunofluorescence is an alternative multiplex IHC solution adopted by the Lunaphore COMET platform (11). These utilize a combination of primary and secondary antibodies that are incubated sequentially to acquire an image followed by antibody stripping. This workflow is repeated several times to generate multiplex IHC data. These platforms enable data interrogation at single cell resolution. Bruker’s GeoMx (12) is an imaging-based spatial proteomics platform that allows users to acquire >1000 protein markers, including post-translational modifications, but is limited to low resolution with a user defined region of interest (ROI) diameter of 50-600 µm. Similar to GeoMx, MALDI is based on imaging mass spectrometry that is a high-throughput, albeit low-resolution, platform.

Several spatial multiomics studies have demonstrated that integrating histopathology with transcriptomic and proteomic layers reveals compartment-specific biology and uncovers emergent molecular programs underlying malignant transformation. CITE-seq combined with multiplex spatial imaging revealed peri-tumoral lymphoid aggregates enriched for B-cell signatures that correlated with improved melanoma immunotherapy outcomes (13). Spatial transcriptomics and proteomics were leveraged to characterize cancer associated fibroblasts (CAFs) across 10 cancer types (14). In low-grade serous carcinoma, such integrated analyses have identified pronounced RNA–protein decoupling and microenvironment-specific signaling networks, underscoring how the spatial context shapes tumor evolution (15). Spatial transcriptomics further highlights that tumor heterogeneity is driven by localized immune infiltration and stromal–immune–tumor crosstalk, revealing spatial gradients of immune effector molecules and cytokine expression that influence disease progression and immunotherapy responsiveness. These high-resolution maps are particularly powerful for detecting cytokines produced by immune or tumor-associated cells, which often act within tightly restricted spatial domains to regulate proliferation, immune evasion, and local inflammation. Additional spatially resolved multiomics analyses in low-grade serous carcinoma have uncovered previously hidden molecular patterns and discrete TME niches, reinforcing the value of integrated RNA–protein spatial mapping for identifying new oncogenic drivers (16). Complementary spatial proteomics studies have mapped protein-level immune signaling at single-cell resolution, revealing cytokine-rich microenvironments and immunosuppressive networks that contribute directly to therapeutic resistance. Recent profiling of solid tumors identified immune-suppressive cell populations and their spatially restricted protein signatures, offering mechanistic evidence of cytokine-mediated resistance within defined tumor niches (17).

Collectively, these studies demonstrate that spatial multiomics are uniquely suited to uncovering cytokine-driven mechanisms of cancer progression. By preserving tissue architecture, these technologies illuminate how cytokine gradients, spatial organization of immune cells and TME-specific signaling cooperatively shape tumor behavior—ultimately enabling more precise and spatially informed therapeutic strategies.

## 2 What spatial systems have already taught us (knowledge base)

Spatial multiomic technologies have already demonstrated their usefulness in a few ways:

- Spatially mapped immune and stromal niches relevant to cytokine biology: Spatial technologies have elucidated gene regulatory networks across cancer types, identified tumor-associated macrophage (TAM) phenotypes predictive of outcome, mapped CAF heterogeneity, and revealed focal environments that drive evolution and therapeutic resistance. For example, IL4I1+ macrophages correlate with favorable prognosis in colon cancer, while NLRP3+ and SPP1+ macrophages mark necrotic territories associated with poor outcomes (18). Spatial mapping of CAFs across cancers shows recurrent patterns of CAF–cancer co-localization or CAF–macrophage co-localization that shape T cell infiltration and cytokine diffusion barriers (19). These niches define microenvironments where cytokine activity is enhanced, sequestered, or neutralized.
- Spatial ligand–receptor topology informs immunocytokine design: Spatial transcriptomics reveals where cytokine receptors accumulate, how they align with T cell, NK cell, and macrophage clustering, and which stromal elements modulate their availability (20). High-resolution maps could show local receptor gradients (e.g., IL-2 receptor subunits CD25/CD122) that guide engineering of receptor-biased variants, and may identify areas where cytokines are immobilized by ECM or scavenged by competing cell types, instructing decisions about linker lengths, affinity tuning, and ECM-anchoring motifs for immunocytokines (21).
- Spatial proteomics resolves RNA–protein mismatches: Because RNA abundance does not necessarily predict protein localization, activation status, or cell–cell distances, high-plex spatial proteomics is critical for validating immunocytokine receptor availability. Spatial proteomics panels measuring >1,000 proteins help identify receptor hotspots, activation epitopes, and potential decoy receptors, enabling more accurate payload selection and receptor-engagement predictions (4, 22).

## 3 Gaps limiting translation of spatial biology into immunocytokine engineering

Despite these breakthroughs, crucial improvements must be made to capitalize on the capabilities of spatial multiomic technologies.

### 3.1 Technical quality control

**Figure 1:**
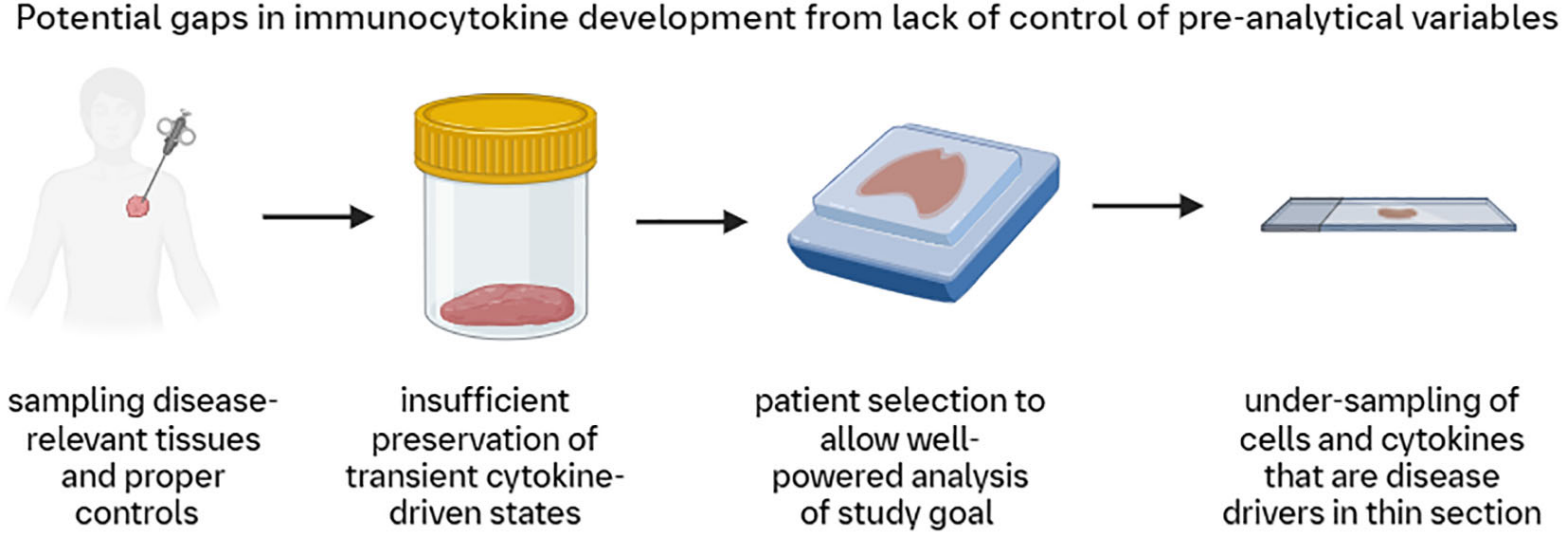
Tissue workflow: following best practices at each step ensures optimal mRNA and protein preservation.

Spatial multiomics techniques on patient tissues provide unparalleled insights into gene and protein expression within tissue architecture, enabling discoveries in disease biology and therapeutic development. However, the complexity of these workflows—from tissue acquisition to data analysis (**Figure 1**) —makes rigorous quality control indispensable. Each step introduces potential variability that can compromise data integrity, reproducibility, and biological interpretation.

Unlike bulk or single-cell transcriptomics, spatial methods rely on intact tissue morphology ensuring the preservation of the spatial context of cells. Poor quality control (QC) at early stages can lead to artifacts that obscure spatial relationships. In addition, spatial assays consume significant resources and are time-consuming. Repeating experiments due to QC failures is costly and delays research. Suboptimal tissue quality and assay conditions negatively affect downstream workflows as errors in tissue preparation or imaging propagate through segmentation, quantification, and analysis, leading to misleading biological conclusions.

To ensure high quality data, QC across the workflow should be included in each step.

#### 3.1.1 Tissue QC

FFPE (formalin-fixed paraffin-embedded) tissue block integrity as well as mRNA and protein preservation are essential for generating high quality spatial omics datasets. Delays or improper fixation of patient tissue material leads to RNA degradation and protein crosslinking artifacts with profound negative effects on spatial data. Instrument vendors established good practice standards to ensure that only high-quality tissues are used to generate spatial biology datasets. Bruker, for example, recommends that tissues are fixed for 18 to 24 hours for tissues of 5 mm thickness with patient material going into fixative immediately post excision for best results (23). Stringent and predictive QC assays should be used before committing samples to spatial workflows, and only material of high quality should be included in dataset generation. Instrument vendors suggest assessing mRNA quality with tests such as DV200 or RIN, but these do not always track with the quality of the generated datasets (24). Using tissues with acceptable mRNA scores according to general guidelines could nevertheless lead to incomplete characterization of cell populations present in patient tissues. Therefore, only tissues close to the maximum of the recommended QC scoring metrics should be considered for dataset generation.

Once suitable tissue samples have been identified from patients selected to ensure well-powered downstream analysis, sections need to be generated with histology and mRNA preservation in mind. When specific glass slide substrates are not provided by the instrument vendor, high quality glass slides capable of retaining the FFPE tissues through the whole process should be selected. Care should be used to ensure that the FFPE tissue sections are placed centrally within the imaging area to avoid edge interferences and that tissue sections stay firmly attached to the glass slides. Tissue nicks or folds should be avoided. The tissue sections should be of even thickness which is suitable for the specific spatial omics technology (generally 4 to 5 µm thick) and the same thickness should be used for the whole study. Additionally, sections should be selected which are representative of all relevant cell types to ensure capturing the cytokine pathways.

#### 3.1.2 Tissue preparation and considerations for instrument parameters

Many spatial transcriptomics technologies integrate antibody-based segmentation markers to ensure proper identification of tissue cells for downstream analysis. Spatial Proteomics technologies equally rely on markers for downstream cell segmentation. This requires proper tissue preparation and digestion to allow for the preservation of protein epitopes while guaranteeing accessibility of the mRNA molecules to the probes for imaging-based spatial transcriptomics methods, all while continuing to avoid mRNA degradation. The best-suited conditions for each tissue type need to be experimentally validated and then consistently applied for each run within a series to avoid batch effects. Once the FFPE sections are loaded onto the instrument, the Region-of-Interest (ROI) is visible for Field-of-View (FoV) selection. Any tissue parts which have detached during the preparation workflow or are visibly out of focus should be avoided for FoV selection. Tissue edges pose a challenge as the FoVs along the outside of tissues contain empty space which artificially reduces QC scores generated per FoV, therefore unnecessary empty spaces should be avoided where feasible. In summary, suboptimal tissue preparation and FoV selection cause loss of signal, with unwanted consequences for the data generated.

### 3.2 Computational limitations

#### 3.2.1 Cell segmentation

Spatial multiomics technologies diverge significantly based on resolution, data acquisition modality, plexity, and sensitivity. For instance, barcode quality for imaging-based spatial transcriptomics, background fluorescence signal of cyclic-IF based spatial proteomics platforms, or batch effects in antibody labelling during imaging mass cytometry experiments; all affect the quality of raw data generated differently. Hence, the data generated using these technologies cannot be processed the same and require some platform specific quality control methods (25). These are highlighted by differing approaches each adopts to segment the cells. Accurately identifying cellular boundaries constitute a fundamental building block in the data deconvolution process and each spatial multiomics technology implements a different solution starting with input data. Visium and Visium HD use H&E images to perform cell segmentation, whereas CosMx, Xenium, or MERFISH, and spatial proteomics platforms such as CODEX, MIBI or COMET, use a fluorescently stained image with a cocktail of antibodies that identify cellular boundaries. Furthermore, each platform has developed custom algorithms that approach cell segmentation in a fundamentally different way (26). Finally, a plethora of publicly available cell segmentation algorithms exist that can be utilized to refine cellular boundaries (27).

#### 3.2.2 Batch corrections

Spatial -omics data often tend to be high plex, sparse, and often geometrically irregular due to inherent tissue heterogeneity complicating the data normalization and batch effect correction process (28). These attributes make data integration a challenge. Batch effects could originate from either biological heterogeneity such as sampling biases and donor cohort, or from non-biological factors such as instruments, imaging, and sequencing. Several batch correction algorithms are designed to mitigate different kinds of batch effects and benchmarking studies have identified Harmony, LIGER, and Seurat as strong performers. However, removing batch effects without over-correcting remains a challenge (29). Recent innovations in this field are occurring on two fronts: Statistical frameworks are developed that can evaluate and inform about the nature of batch effects that exist in a dataset (30, 31) and concurrently, novel algorithms are specifically tailored to spatially resolved multiomics data (26). Collectively, these factors invariably introduce technology specific biases or errors, which are then amplified and propagated to affect the interpretability and reproducibility of downstream analyses.

#### 3.2.3 Niche reconstruction

Tissue homeostasis emerges from an intricate interplay of molecular and cellular processes that collectively sustain organ function. A central challenge in modern biology is understanding how single cells assemble into spatially organized niches and activate context-dependent gene expression and cell–cell communication programs that shape tissue architecture and influence disease progression. Spatially resolved multiomics recreate the tissue architecture by preserving location of cells. However, the application of spatial niche frameworks is required to reveal insights into disease mechanisms. The computational discovery of spatial niches has advanced rapidly. Early methods relied on fixed nearest-neighbor windows (typically 10–200 cells) to infer local cellular composition (32). These approaches were effective in spatial proteomics, where antibody panels limited phenotyping complexity, but they introduced biases and errors when applied to high-dimensional spatial transcriptomics data. Subsequent probabilistic and Bayesian models—such as HMRF, BayesSpace, and DRSC—integrated gene expression with spatial adjacency to assign cells to niches via soft boundaries based on the Potts model (33–35). Although powerful, these approaches often struggled in highly heterogeneous tissues where neighboring cells did not necessarily share phenotypic similarity. The BASS algorithm improved upon this by integrating hierarchical modeling to capture gene expression alterations across spatial domains (36).

**Figure 2:**
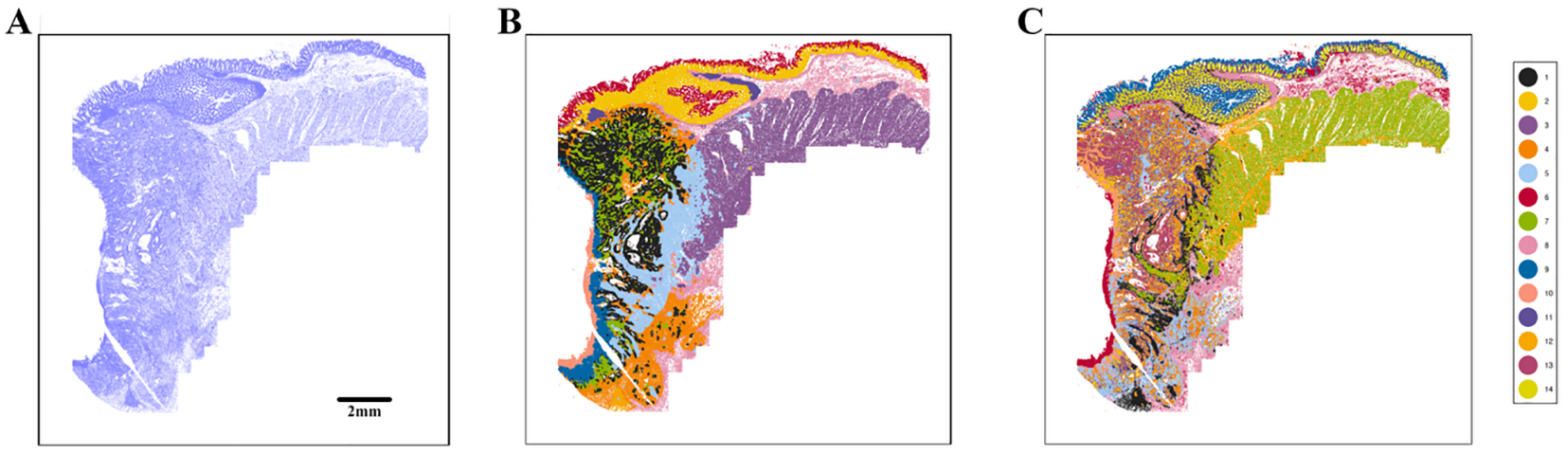
Spatial niche clustering algorithms were applied to the publicly available Colon FFPE tissue dataset generated by Bruker on CosMx SMI. **(A)** Spatial recreation of the tissue. **(B, C)** Spatial niche map generated by either **(B)** BANKSY or **(C)** Cell Charter algorithms. The image represents notably different spatial niche maps generated depending on the algorithm. (Data source: https://brukerspatialbiology.com/products/cosmx-spatial-molecular-imager/ffpe-dataset/cosmx-human-whole-transcriptome-colon-dataset/).

A new generation of algorithms employs graph-based and deep learning strategies—including SCGP, SpaGCN, STAGATE, GraphST, Giotto, UTAG, CellCharter, BANSKY and scNiche—to construct cellular networks using nearest-neighbor or radius-based distances (typically 50–200 μm). These models incorporate gene expression, spatial topology, and even histological imagery to resolve fine-grained niches (21, 24, 37–42). Still, methodological choices such as network construction, radius selection, and dimensionality reduction can strongly influence niche detection (**Figure 2**). Genes with broad or high expression, especially lineage markers, may overshadow subtle state transitions, motivating complementary analytical approaches like spatial autocorrelation methods (InSituCor, Hotspot) that detect localized gene modules independent of cell identity (43, 44).

Benchmarking studies highlight that no single method performs optimally across all tissue types, resolutions, or platforms (45). Consequently, integrating spatial computational frameworks with expert histopathological interpretation remains vital. As spatial multiomics continues to expand, establishing standardized, hierarchical definitions of spatial niches will be essential for generating reproducible insights and linking microscale molecular events with macroscopic tissue pathology.

### 3.3 Other limitations

#### 3.3.1 Cybersecurity

Working with spatial -omics data raises IT and cybersecurity-related challenges due to the sheer scale and volume. These technologies generate highly heterogeneous data ranging from sequencing to high-resolution imaging data; each with their bespoke data generation and handling mechanisms. They produce inherently incompatible file formats, metadata schemas, and spatial coordinates. Furthermore, they employ data storage solutions that range from local server to cloud storage with ecosystems built to automate cell segmentation and image analysis. In such a scenario, just consolidating data and preventing data fragmentation becomes a formidable IT challenge requiring implementation of novel infrastructure that is flexible to accommodate future innovations (46, 47).

Analyzing and integrating multi-omics data requires cloud based high performance computing (HPC) environments with scalable architecture that can execute GPU- and RAM-intensive tasks such as building graph-based networks, cell segmentation, multi-modal data integration, etc. In addition, cloud-based HPC also provides version control and provenance tracking, a critical aspect of the high dimensional data analysis process. Consolidating and analyzing these data also raises cybersecurity issues in protecting patient metadata with the need for rigorous security measures such as controlled access, secure API controls, and certificate-based authentication on HPC. Special attention should also be paid to mitigate issues related to unsecured S3-compatible storage, API token leakage, and lateral data movement across networks if they do not implement zero-trust principles. Protecting these datasets requires a multilayered strategy combining encryption at rest and in transit, federated identity management, hardware-level security modules, audit-ready logging, and rigorous vendor compliance frameworks. Only with these safeguards can spatial multiomics data be shared efficiently across research, clinical, and industry environments without compromising patient privacy or the integrity of analytical outcomes (48).

## 4 Implications for immunocytokine engineering

With spatial multiomics technology working with improved technical QC and computational workflows, there are a few key areas where immunocytokine engineering are poised to benefit with improved engineering outcomes. As outlined in Section 3, rigorous tissue QC and accurate cell segmentation preserve spatial architecture, minimize transcript dropout, and resolve true cellular boundaries, enabling spatial datasets to inform quantitative engineering constraints rather than qualitative observations.

### 4.1 Targeted delivery design

Spatial maps reveal cytokine receptor expression patterns, guiding selection of targeting domains for antibody–cytokine fusion proteins. Designs such as ECM-targeted L19–IL2 illustrate how localized engagement enhances therapeutic effect and minimizes systemic diffusion (49, 50). High-resolution maps (e.g., Stereoseq) validate receptor-rich microdomains as priority engagement sites. In addition, QC-driven niche reconstruction enables accurate measurement of cell–cell synapse distances between antigen-expressing cells and cytokine-responsive immune cells, providing spatial constraints that inform linker length and flexibility for effective bridging (51). Poor QC, by contrast, can distort spatial relationships and misrepresent proximity, leading to suboptimal targeting geometries (52).

### 4.2 Overcoming niche competition

Spatial transcriptomics uncovers cytokine sinks—Treg-dense regions, CAF-mediated exclusion zones, and suppressive myeloid clusters—that immunocytokines must circumvent. Protease-activatable IL-12 analogs (masked IL-12Fc) restrict activity to protease-rich tumor environments, improving therapeutic index and limiting systemic toxicity (53, 54). With robust QC minimizing transcript dropout, spatial datasets can also resolve low-level receptor expression in non-target tissues, improving prediction of off-target sink liabilities (17). Furthermore, accurate spatial resolution reveals regulatory elements such as decoy receptors or scavenger receptors, which modulate cytokine availability and signaling, enabling engineering strategies that avoid sequestration and preserve cytokine activity (55, 56).

### 4.3 Selecting novel cytokine targets

Spatial secretome analyses identify non-canonical cytokines (e.g., IL-33, CXCL12) enriched in particular CAF–immune interfaces, which may be incorporated into next-generation immunocytokine payloads (17). Critically, QC-dependent improvements in resolution and segmentation allow distinction between true intercellular signaling and technical bleed-through from adjacent cells, ensuring that identified ligand–receptor interactions reflect biologically relevant communication networks suitable for therapeutic exploitation (57, 58).

### 4.4 Region-specific efficacy profiling and dosing

Spatial remodeling following cytokine exposure (e.g., IL-12 cytokine factories) defines where sustained activity should be targeted. Timed dosing after checkpoint inhibitors may amplify effector niches that were absent pre-therapy. High-quality QC further enables quantitative assessment of target density and subcellular localization, distinguishing membrane-accessible targets from intracellular pools (17, 59). These measurements inform affinity optimization:

- In high target density niches, lower-affinity designs can exploit avidity to preferentially bind clustered pathological regions.
- In low target density niches, higher-affinity or multivalent formats may be required to ensure sufficient cytokine delivery.

Collectively, these examples highlight how deficiencies in tissue QC and segmentation propagate into erroneous spatial assumptions that directly impact immunocytokine design decisions, underscoring the dependence of therapeutic engineering on data fidelity.

### 4.5 Case study: L19–IL2 in PDAC

A recent study on pancreatic ductal adenocarcinoma (PDAC) illustrates this impact. Researchers investigated the efficacy of an immunocytokine, L19–IL2, designed to deliver interleukin-2 selectively to tumors via the L19 antibody targeting EDB-fibronectin (33, 60). Stereoseq was employed to map immune cell infiltration and activation within the TME following treatment. Spatial analysis revealed that L19–IL2 remodeled immunologically ‘cold’ tumors into ‘hot’ ones by recruiting CD8+ T cells, NK cells, and antigen-presenting cells into the tumor core, alongside upregulation of IL-2 receptor genes and cytotoxic markers (e.g., granzymes, perforins). These findings, validated by Stereoseq clustering and heatmaps, demonstrated how spatial transcriptomics can uncover localized immune activation patterns that bulk RNA-seq alone cannot resolve.

## 5 Conclusion

Spatial multiomics—when grounded in rigorous QC and thoughtful computational frameworks—provides a mechanistic lens through which the immune architecture of tumors can be interrogated with unprecedented clarity. This integration of spatial biology with immunocytokine engineering represents a transformative shift: therapies can now be designed to exploit, remodel, or reprogram defined immune ecosystems rather than relying on systemic cytokine exposure. Embedding spatially informed principles into cytokine therapy development will accelerate the creation of targeted, safer, and more effective immunocytokine therapies capable of reshaping the TME and improving patient outcomes.
